# Effects of Sponge-Derived Alkaloids on Activities of the Bacterial α-D-Galactosidase and Human Cancer Cell α-N-Acetylgalactosaminidase

**DOI:** 10.3390/biomedicines9050510

**Published:** 2021-05-05

**Authors:** Natalia Utkina, Galina Likhatskaya, Olesya Malyarenko, Svetlana Ermakova, Larisa Balabanova, Lubov Slepchenko, Irina Bakunina

**Affiliations:** G.B. Elyakov Pacific Institute of Bioorganic Chemistry, Far Eastern Branch, Russian Academy of Sciences, Vladivostok 690022, Russia; utkinan@mail.ru (N.U.); galin56@mail.ru (G.L.); vishchuk@mail.ru (O.M.); swetlana_e@mail.ru (S.E.); lbalabanova@mail.ru (L.B.); lubov99d@mail.ru (L.S.)

**Keywords:** marine sponges, aaptamines, makaluvamines, slow-binding irreversible inhibitors, bacterial α-D-galactosidase, cancer α-N-acetylgalactosaminidase

## Abstract

During a search for glycosidase inhibitors among marine natural products, we applied an integrated in vitro and in silico approach to evaluate the potency of some aaptamines and makaluvamines isolated from marine sponges on the hydrolyzing activity of α-N-acetylgalactosaminidase (α-NaGalase) from human cancer cells and the recombinant α-D-galactosidase (α-PsGal) from a marine bacterium *Pseudoalteromonas* sp. KMM 701. These alkaloids showed no direct inhibitory effect on the cancer α-NaGalase; but isoaaptamine (**2**), 9-demethylaaptamine (**3**), damirone B (**6**), and makaluvamine H (**7**) reduced the expression of the enzyme in the human colorectal adenocarcinoma cell line DLD-1 at 5 μM. Isoaaptamine (**2**), 9-demethylaaptamine (**3**), makaluvamine G (**6**), and zyzzyanone A (**7**) are slow-binding irreversible inhibitors of the bacterial α-PsGal with the inactivation rate constants (*k*_inact_) 0.12 min^−1^, 0.092 min^−1^, 0.079 min^−1^, and 0.037 min^−1^, as well as equilibrium inhibition constants (*K_i_*) 2.70 µM, 300 µM, 411 µM, and 105 µM, respectively. Docking analysis revealed that these alkaloids bind in a pocket close to the catalytic amino acid residues Asp451 and Asp516 and form complexes, due to π-π interactions with the Trp308 residue and hydrogen bonds with the Lys449 residue. None of the studied alkaloids formed complexes with the active site of the human α-NaGalase.

## 1. Introduction

Marine organisms, in particular marine sponges, are inexhaustible source of metabolites with various biological activities. A large group of secondary metabolites isolated from marine sponges are alkaloids [1]. Marine sponges of the genera *Latrunculia*, *Batzella*, *Prianos*, *Zyzzya* are a rich source of alkaloids bearing a pyrrolo[4,3,2-*de*]quinoline skeleton [2]. Pyrroloquinoline alkaloids have shown a variety of biological activities, including antifungal and antimicrobial [3], antioxidant [4], antimalarial activity [5]; inhibition of the HIF-1α/p300 interaction [6]. Some makaluvamines demonstrated in vitro and in vivo cytotoxicity against various types of cancer [7,8], which was attributed to the inhibition of topoisomerase II [9]. It is shown that numerous pyrroloiminoquinone alkaloids, including synthetic analogues, are active, their mechanisms of action, pharmacological properties, and safety have shown that they have great potential for creating new anticancer drugs [10].

The next interesting small group of biologically active marine alkaloids having a rare 1*H*-benzo[*de*]-1,6-naphthyridine skeleton are aaptamines [11]. Aaptamines have been isolated from marine sponges mainly of the genus *Aaptos*. All these compounds have either an N-methylated or a non-N-methylated 1,6-naphthyridine core fused with a functionalized benzenoid unit. Some of aaptamines have been reported to have α-adrenoceptor blocking [12], anti-HIV-1 [13,14], antimycobacterial [13,14,15], antiherpes [16], and anticancer activity [17]. Aaptamine and its congeners were shown to act as inhibitors of different enzymes. They inhibit sortase A [18], monoamine oxidase [19], β-1,3-glucanase of marine mollusks [20], and showed proteasomal inhibitory activity [21].

In the last years, marine organisms have received increased attention as a source of enzyme inhibitors, due to their potential use in pharmacology [22]. Enzyme inhibitory activity is often found in sponge metabolites, among which kinase inhibitors are the most representative class of isolated inhibitors [23]. Glycosidase-catalyzed carbohydrate hydrolysis is a biologically widespread process. An imbalance in glycosidase functions has been considered one of the causes of some diseases, including cancer, viral infections, and metabolic and genetic disorders [24,25,26,27,28]. However, relatively few attempts have been made to find inhibitors of glycoside hydrolases, such as α-D-galactosidases (α-D-galactoside galacto hydrolases, EC 3.2.1.22) and α-N-acetylgalactosaminidases (EC 3.2.1.49) among huge number of structurally unique low molecular marine natural products.

The enzyme α-D-galactosidase from the cold-adaptable marine bacterium *Pseudoalteromonas* sp. KMM 701 is a typical exo-glycosidase that cleaves the α-D-galactopyranos residues from the nonreducing end of various oligosaccharides including α-1,3-bound galactose residues of glycoprotein of group substance of red blood B-cells [29]. It belongs to the 36 family of glycoside hydrolases (GH) and has research interests, due to various possible fields of application, such as blood transfusion, infection diseases, structural studies in glycobiology [30,31]. α-N-Acetylgalactosaminidase, which removes terminal α-linked residues of N-acetylgalactosaminide, is widespread in organs and tissues of mammals, bacteria, and fungi, is produced by all cancer cells and accumulated in the blood plasma of cancer patients, leading to immunosuppression in patients with advanced cancer [32,33]. Cancer α-NaGalase belongs to the GH27 family. The level of enzyme activity and number its forms increase in blood serum, especially at the initial stage of the disease and the stage of metastasis [34]. Thus, α-N-acetylgalactosaminidase, being an immunosuppressive agent in cancer patients, is considered as a potential therapeutic target in cancer treatment.

It is known that glycosidase inhibitors can regulate or block the enzyme imbalance [24]. Therefore, the identification of potential inhibitors of both α-N-acetylgalactosaminidase and α-D-galactosidase and their mode of action is of importance. Galacto-iteamine, unnatural analog of alkaloid iteamine (o-aminobenzyl β-D-glucopyranoside) isolated from *Itea virginica* L. inflorescence was the weak direct inhibitor of chicken liver α-N-acetylgalactosaminidase [35]. It was shown that the iminosugar DGJNAc can inhibit, stabilize, and chaperone human α-NAGAL both *in vitro* and in vivo [36], but fucoidan from *Fucus evanescens* did not inhibit free α-NaGalase, however, it reduced the expression of the enzyme in the DLD-1 cells [37]. The previously characterized low molecular inhibitors of the α-D-galactosidase from marine sources are polyhydroxynaphthoquinone pigments from sea urchins and their synthetic analogues [38], polybrominated diphenyl ether and dibenzo-*p*-dioxins from *Dysidea* sponges [39] and pentacyclic guanidine alkaloids from the sponge *Monanchora pulchra* [40].

The main goal of this study was to evaluate in vitro alkaloids aaptamine (**1**), isoaaptamine (**2**), 9-demethylaaptamine (**3**), aaptanone (**4**), damirones A (**5**) and B (**6**), makaluvamines H (**7**) and G (**8**), and zyzzyanone A (**9**) isolated from marine sponges as direct or indirect inhibitors of α-N-acetylgalactosaminidase (α-NaGalase) from human cancer cells. Herein, evolutionarily related glycoside hydrolase α-D-galactosidase from the marine bacterium *Pseudoalteromonas* sp. KMM 701 was used in addition to compare their enzyme inhibitory potential, and explore their possible way of interacting with enzymes through kinetic and molecular docking studies.

## 2. Materials and Methods

### 2.1. Materials and Reagents

Roswell Park Memorial Institute Medium (RPMI 1640), phosphate buffered saline (PBS), l-glutamine, penicillin-streptomycin solution, trypsine and fetal bovine serum (FBS), sodium hydrocarbonate (NaHCO_3_), and agar were purchased from BioloT (Bolshoy Sampsonievsky avenue, Saint-Petersburg, Russia), p-nitrophenyl-N-acetyl-α-D-galactosaminide (p-NPNA-α-Gal), p-nitrophenyl-α-D-galactopyranoside (pNP-α-Gal), and Bradford reagent were purchased from Sigma-Aldrich (St. Louis, MO, USA); recombinant protein markers SDS-PAGE-electrophoresis, from BioRad (1000 Alfred Nobel Drive, Hercules, CA, USA). Silica gel (Sorbpolymer, Krasnodar, Russia), Sephadex LH-20 (Pharmacia Fine Chemicals), Polyamide (Woelm, Germany), and Polychrom-1 (powdered Teflon, Biolar, Olaine, Latvia) were used for column chromatography, Sorbfil plates coated with silica gel F254 (Sorbpolymer, Krasnodar, Russia) were used for TLC.

### 2.2. Experimental Equipment

Microplate spectrophotometer (BioTek Instruments, Highland Park, Winooski, VT, USA) was used for measuring of optical density at 400 nm (D400). Ultrasonic homogenizer Bandelin Sonopuls (Bandelin electronic GmbH & Co., KG Heinrichstraße 3–4 12, 207, Berlin, Germany) was used for homogenization. GenBAflex-tubes 6–8 kDa (Scienova GmbH, Wildenbruchstabe 15 07745 Jena, Germany) were used for dialysis. 1H NMR and 13C NMR spectra were recorded on a Bruker AVANCE DRX-500 NMR spectrometer at 500 and 125 MHz, respectively. High-resolution electron ionization mass spectrometry (HREI MS) and high-resolution fast atom bombardment mass spectrometry (HRFAB MS) were performed on an AMD-604 S mass spectrometer (Intectra, Germany).

### 2.3. Collection and Identification of Sponge Material

We used freeze-dried samples of sponges *A. aaptos* (O45-113) and *Z. fuliginosa* (O9-407), collected during scientific cruises of R/V Academik Oparin, which are kept in the collection of G.B. Elyakov Pacific Institute of Bioorganic Chemistry, Far Eastern Branch, Russian Academy of Sciences. The sponge *A. aaptos* was collected at Tho Chu Island (9°18.5′ N 103°29.6′ E, at a depth of 6 m), South China Sea, Vietnam, in May 2013 during the 45th scientific cruise of R/V Academik Oparin. The sponge *Z. fuliginosa* was collected at Mid Islet (16°14′ S 149°59′ E, at a depth of 10 m), Eastern Australia, in July 1989 during the 9th scientific cruise of R/V Academik Oparin. Sponge samples were freeze-dried and stored in a refrigerator until used. The sponges were taxonomically identified by Dr. Vladimir B. Krasokhin.

### 2.4. Isolation and Purification of Compounds

A freeze-dried sample of the sponge *A. aaptos* (20 g) was extracted exhaustively with acetone, followed by EtOH. The acetone extract was chromatographed on a silica gel column in CHCl_3_–EtOH (3:2) to obtain aaptamine (**1**; 6 mg, yield 0.03% on dry weight of sponge), isoaaptamine (**2**; 3.8 mg, yield 0.019%), and 9-demethylaaptamine (**3**; 3.5 mg, yield 0.017%) after purification on a Sephadex LH-20 column in CHCl_3_–EtOH (3:1). The EtOH extract was concentrated and chromatographed on a Polyamide column with EtOH to give aaptanone (**4**; 3 mg, yield 0.015%), final purification was achieved on a Sephadex LH-20 column using 50% aqueous EtOH. Compounds **1**–**4** were identified by comparison of their spectroscopic data with those reported earlier [12,14,41,42], respectively.

Aaptamine (**1**): HREI MS *m/z* 229.0973 [M+H]^+^, (calcd. for C_13_H_13_N_2_O_2_: 229.0977);

Isoaaptamine (**2**): HREI MS *m/z* 229.0971 [M+H]^+^, (calcd. for C_13_H_13_N_2_O_2_: 229.0977);

9-demethylaaptamine (**3**): HREI MS *m/z* 215.0817 [M+H]^+^, (calcd. for C_12_H_11_N_2_O_2_: 215.0820);

Aaptanone (**4**): HRFAB MS *m/z* 281.0541 [M+Na]^+^, (calcd. for C_13_H_10_N_2_O_4_Na: 281.0538).

A freeze-dried sample of the sponge *Z. fuliginosa* (50 g) was extracted with 50% EtOH at room temperature; the solvent was concentrated to yield a dark red residue. This residue was triturated with CHCl_3_ to obtain the CHCl_3_-solubles, which were chromatographed on a Sephadex LH-20 column in CHCl_3_ to obtain damirones A (**5**; 22 mg, yield 0.044%) and B (**6**; 18 mg, yield 0.036%). A CHCl_3_-insoluble solid was subjected to column chromatography on a Polychrome-1 column with a solvent elution gradient from H_2_O to EtOH. Dark red fractions (25–40% EtOH) were concentrated and chromatographed on a Sephadex LH-20 column in CHCl_3_-EtOH-TFA (4:1:0.1%) to obtain makaluvamine H (**7**; 8.5 mg, yield 0.017%). A brownish-green fraction (50% EtOH) was repeatedly chromatographed on a Sephadex LH-20 column in CHCl_3_-EtOH-TFA (3:1:0.1%) to obtain makaluvamine G (**8**; 27.5 mg, yield 0.055%) and zyzzyanone A (**9**; 3 mg, yield 0.006%). Compounds **5**–**9** were identified by comparison of their spectroscopic data with those described earlier [43,44,45,46], respectively.

Damirone A (**5**): HREI MS *m/z* 216.0895 [M]^+^, (calcd. for C_12_H_12_N_2_O_2_: 216.0899);

Damirone B (**6**): HREI MS *m/z* 202.0739 [M]^+^, (calcd. forC_11_H_10_N_2_O_2_: 202.0742);

Makaluvamine H (**7**): HREI MS *m/z* 217.1211[M+H]^+^, (calcd. for C_12_H_15_N_3_O: 217.1215),

Makaluvamine G (**8**): HREI MS *m/z* 334.1550 [M+H]^+^, (calcd. for C_20_H_20_N_3_O_2_: 334.1555),

Zyzzyanone A (**9**): HRFAB MS *m/z* 350.1500 [M+H]^+^, (calcd. for C_20_H_20_N_3_O_3_: 350.1504).

### 2.5. Cell Cultured

Cancer cells RPMI-7951 (ATCC^®^ no. HTB-66™), MDA-MB-231 (ATCC^®^ no.HTB-26™), DLD-1 (ATCC^®^ no.CCL-221), HT-29 (ATCC^®^ no.HTB-38™), HCT-116 (ATCC^®^ no.CCL-247), SK-MEL-5 (ATCC^®^ no.HTB-70™), and mouse healthy epidermal cells JB6 Cl41 (ATCC^®^ no. CRL-2010™) were obtained from the American Type Culture Collection (Manassas, VA, USA). Cells were cultured in appropriate culture medium supplemented with 10% FBS and 1% penicillin–streptomycin solution. The cell cultures were maintained at 37 °C in humidified atmosphere containing 5% CO_2_. For pretreatments with alkaloids human colorectal adenocarcinoma cell line DLD-1 was cultured in the same RPMI-1640 medium supplemented with 10% fetal bovine serum (FBS) and penicillin–streptomycin solution. Cells were maintained in a sterile environment and kept in an incubator at 5% CO_2_ and 37 °C to promote growth. DLD-1 cells were sub-cultured every 3–4 days by their rinsing with phosphate-buffered saline (PBS), adding trypsin to detach the cells from the tissue culture flask, and transferring 10–20% of the harvested cells to a new flask containing fresh growth media.

### 2.6. Cytotoxic Activity Assays

DLD-1 cells (8 × 10^3^/200 μL) were seeded in 96-well plates for 24 h at 37 °C in a 5% CO_2_ incubator. The cells were treated with compounds **1**–**9** at concentrations ranging from 0 to 20 μM for an additional 24 h. Subsequently, cells were incubated with 15 μL MTS (3-(4,5-dimethylthiazol-2-yl)-5-(3-carboxymethoxyphenyl)-2-(4-sulfophenyl)-2H-tetrazolium) reagent for 3 h, and the absorbance in each well was measured at 490/630 nm using a microplate reader “Power Wave XS” (Bio Tek, Winooski, VT, USA). All the experiments were repeated three times, and the mean absorbance values were calculated. The results are expressed in absorbance by the compound’s treatment compared to the non-treated cells (control 1) and the compounds in medium (control 2). All the experiments were made in triplicate.

### 2.7. Cell Treatment with Alkoloids

DLD-1cells (5.0 × 10^5^) were seeded in 6 mm dishes for 24 h at 37 °C in a 5% CO_2_ incubator. Then the cells were treated with compounds **1**–**9** at concentrations ranging from 0 to 20 μM for an additional 24 h. Then the cells were harvested using 0.02% EDTA, after this 3 mL of cold 15 mM Tris–HCl (pH 7.0) was added.

### 2.8. Preparation of Cell Lysate

Every 3–4 days cells were rinsed in phosphate buffered PBS, detached from the tissue culture flask by 1× trypsin/EDTA solution, harvested with RPMI-1640 medium and centrifuge at 500 rpm for 3 min. The culture media was discarded and cells pellet was resuspended in EDTA/Tris solution and frozen at −80 °C.

### 2.9. Isolation and Purification of α-N-Acetylgalactosaminidase from Cell Lysates

Frozen lysates of cancer cells in the 15 mM Tris buffer, pH 7.0 containing 0.02% EDTA, defrosted and sonicated 10 times during 1 min with a break of 1 min. To remove the cellular detritus, the cell homogenate was centrifuged at 4 °C, 10,000 rpm, 30 min. The supernatant proteins were precipitated with 35% ammonium sulfate, kept during four hours at 4 °C to form a pellet. The protein pellet was collected by centrifugation (4 °C, 10,000 rpm, 30 min) and dissolved in 0.05 M sodium citrate buffer, pH 5.0. The supernatant proteins were precipitated with 75% ammonium sulfate, kept overnight at 4 °C to form a pellet. The protein pellet was collected by centrifugation (4 °C, 10,000 rev/min, 30 min) and dissolved in 0.05 M sodium citrate buffer, pH 5.0. Both extracts were dialyzed against the same buffer, centrifuged to separate the insoluble precipitate. α-NaGalase activity was tested in both extracts and was found only in the protein fraction precipitated with 75% ammonium sulfate. This supernatant was used in further work as partly purified enzyme. The purification quality was controlled by 12% Laemmli-SDS-PAGE-electrophoresis [47].

### 2.10. Production and Purification of Recombinant α-D-galactosidase

The recombinant wild-type α-D-galactosidase (α-PsGal) was produced as described earlier [48]. The plasmid DNA pET-40b(+) containing insertion of the gene from the marine bacterium *Pseudoalteromonas* sp. KMM 701 encoding α-PsGal was transformed in the *Escherichia coli* strain Rosetta (DE3). Heterological expression was carried out at optimal conditions as described earlier [49]. Purification of the recombinant α-PaGal was performed, according to described procedures [48]. The purification quality was controlled by 12% Laemmli-SDS-PAGE-electrophoresis [47].

### 2.11. Enzyme Assay

The activity of α-PsGal and α-NaGalase were determined by increasing the amount of p-nithrophenol (pNP). To assay the α-NaGalase activity 0.05 mL of cell extract and 0.1 mL of substrate pNPNA-α-Gal (8.8 mM) in 0.05 M sodium citrate buffer, pH 5.0 were placed in cells of 96-well plate and incubated at 37 °C for 30 min. The mixtures containing 0.05 mL of an enzyme solution and 0.1 mL of a substrate solution (3.3 mM) in 0.05 M sodium phosphate buffer (pH 7.0) were incubated at 20 °C during 5 min for determination of α-PsGal standard activity. The reactions were stopped by the addition of 0.15 mL of 1 M Na_2_CO_3_ solution.

Absorbance was measured at 400 nm. Results were read with a computer program Gen5 and treated with ExCel computer program. The unit of standard activity (U) was defined as the amount of the enzyme catalyzing the formation of 1 µmol of p-nitrophenol (ε_400_ = 18,300 M^−1^ cm^−1^) per 1 min under the conditions indicated. Specific activity (A) was calculated as the enzyme activity (U) per 1 mg of protein. All calculations were based on reactions with consumption of 10% of the chromogenic substrate. The protein concentrations were estimated by the Bradford method with BSA as a standard [50]. Residual activity (%) was calculated with formula: Residual activity (%) = *v/v*_0_ ×100, where *v* is the initial reaction rate of enzymes under the action of an inhibitor or other factors, *v*_0_ is the initial reaction rate of enzymes in the absence of the influence of any factors. Buffer solutions of α-PsGal (0.1 U/mL) and α-NaGalase (0.01 U/mL) were used in the further experiments.

### 2.12. The Inhibitory Potency of the Compounds **1**–**9** for the α-PsGal and α-NaGalase

To preliminary test the inhibitory potency of the compounds 25 μL the enzyme solutions of α-PsGal in 0.05 M sodium phosphate buffer (pH 7.0) or α-NaGalase in 0.05 M sodium citrate buffer (pH 5.0) were preincubated for 25 min at 20 °C with 5 μL of water soluble or 2 μL of ethanol soluble test compounds at various concentrations, respectively (from 10^−3^ M to 10^−7^ M in a probe), to allow enzyme and inhibitor interaction. In the control sample, 5 μL of water or 2 μL of EtOH were added to 25 μL of enzyme solution. The enzyme reaction was initiated by adding 120 μL of the substrate pNP-α-Gal (3.3 mM) for α-PsGal or pNPNA-α-Gal (8.8 mM) for α-NaGalase in the respective buffer solutions. After 10 min for α-PsGal or 60 min for α-NaGalase the reaction was stopped by adding 150 μL of 1 M Na_2_CO_3_. The amount of pNP was quantified by spectrophotometric detection at 400 nm. Results were evaluated as the percent of residual activity as described above.

### 2.13. The Irreversibility of α-PsGal Inhibition by Alkaloids

To determine the reversibility of the α-PsGal activity inhibition by alkaloids **2**, **3**, **8**, and **9** 15 µL of 0.625 mM aqueous solution of the compounds **2** and **3** or 6 µL of 2.5 mM ethanol solution of the compound **8** and **9** were added to 75 µL of the enzyme solution in 0.05 M sodium phosphate buffer (pH 7.0). After 30 min of the mixture incubation the aliquot of 30 µL was taken and 120 µL of 3.3 mM pNPG solution were added to initiate the reaction; the reaction was stopped by addition of 150 µL of 1M Na_2_CO_3_. The α-D-galactosidase activity was determined, as described above. The remaining reaction mixture was dialyzed against 1 L of 0.02 M sodium phosphate buffer (pH 7.0) for 60 h at 4 °C. The buffer was changed 3 times during the dialysis. The enzyme activity was determined, as described above. A sample of α-PsGal treated with H_2_O or EtOH in the absence of the inhibitor (75 µL of the enzyme solution and 15 µL of H_2_O or 6 µL of EtOH, respectively) was dialyzed and used as a control for determination of the initial activity. The experiment was carried out in two replicates. The residual activity was calculated as described above.

### 2.14. Kinetic Studies on **2**, **3**, **8** and **9** against the α-PsGal

The kinetic parameters *K_i_* and *k*_inact_ were determined by classic methods [51]. The inactivation of α-PsGal was monitored by incubation of the enzyme with different concentrations of alkaloids in 0.02 mM sodium phosphate buffer, pH 7.0, at 20 °C in total volume of 30 µL (25 µL and 5 µL of α-PsGal and alkaloids, respectively), which were placed in cells of 96 cells plate. After preincubation of enzyme-inhibitor mixture for 0.5, 5, 10, 15, 20, 25, or 30 min 120 µL the fraction of remaining enzyme activity was measured as a function of time by addition to the inactivation mixture 120 µL of a solution of pNP-α-Gal (3.3 mM). The pseudo-first-order rate constant of inactivation (*k*_obs_) was determined for each inactivator concentration as the slope of the v/v_0_ dependence on the incubation time in semilogarithmic coordinates. The Excel software was used for these calculations. The second order rate constants for the inactivation process were determined by fitting the dependences of the *k*_obs_ values on the concentration of the inactivators to the Hill or Michaelis–Menten equations. An analysis of the graphs and the choice of models for calculation of inhibition constants (*K_i_*, mM) and inactivation rate constants (*k*_inact_, min^–1^) were performed with the Origin computer software.

### 2.15. Theoretical Models of α-D-Galactosidase Complexes with Aaptamine and Makaluvamine Alkaloids

The 3D model of α-D-galactosidase (GenBank: ABF72189.2) was built using the Molecular Operating Environment version 2020.09 package (MOE, 2020.09; Chemical Computing Group ULC, 1010 Sherbrooke St. West, Suite #910, Montreal, QC, Canada, H3A 2R7, 2020), as described earlier [39,40]. Structures of alkaloids were obtained from the PubChem database and optimized using the MOE program. The evaluation of structural parameters, contact structure analysis, molecular docking, and visualization of the results were carried out with the Dock and Ligand interaction modules in the MOE 2020.09 program. The results were obtained using the equipment of Shared Resource Center Far Eastern Computing Resource of Institute of Automation and Control Processes Far Eastern Branch of the Russian Academy of Sciences (IACP FEB RAS, available online: https://cc.dvo.ru (accessed on 30 November 2020).

## 3. Results and Discussion

### 3.1. Identification of the Compounds

For the present study, alkaloids were reisolated from repository samples of marine sponges *Aaptos aaptos* and *Zyzzya fuliginosa*. Aaptamine (**1**) [12], isoaaptamine (**2**) [14], 9-demethylaaptamine (**3**) [41], and aaptanone (**4**) [41] were reisolated from a sample of the marine sponge *A. aaptos*. Damirones A (**5**) and B (**6**) [43], makaluvamines H (**7**) [44] and G (**8**) [45], and zyzzyanone A (**9**) [46] were reisolated from a sample of the marine sponge *Z. fuliginosa*. Compounds **1**–**9** were identified by comparison of their spectroscopic data (^1^H-, ^13^C-NMR, HREI MS, HRFAB MS) with published values [12,14,41,42,43,44,45,46], respectively. Structures of these alkaloids are shown in Table 2. These compounds present two classes of alkaloids. Aaptamine class includes aaptamine (**1**), isoaaptamine (**2**), and 9-demethylaaptamine (**3**), having 1,6-naphthyridine core fused with a functionalized benzenoid unit, and aaptanone (**4**), a zwitterionic metabolite bearing two adjacent carbonyl groups in the 1,6-naphthyridine core. Makaluvamine class includes damirones A (**5**) and B (**6**), makaluvamines H (**7**) and G (**8**) with a pyrrolo[4,3,2-*de*]quinoline core, and related zyzzyanone A (**9**) bearing dipyrroloquinone core.

### 3.2. Cytotoxic Effect of Alkaloids on Human Colorectal Adenocarcinoma Cell Line DLD-1

We have previously shown that the DLD-1 adenocarcinoma cell line expresses α-NaGalase better than other cell lines, and this enzyme was well characterized [37]. Therefore, for research the effect of alkaloids on the enzyme expression, we used the cell line DLD-1. We investigated the effect of sponge-derived alkaloids on α-NaGalase activity in human cancer cells using two assays: one was a treatment of DLD cells with alkaloids (in vitro); the other was a direct treatment with alkaloids of isolated and purified α-NaGalase from lysates of various cancer cell lines.

As a first step, the effect of compounds **1**–**9** on the viability of human colorectal carcinoma DLD-1 cells was tested by MTS assay to determine their non-toxic concentration. It was shown that compounds **1**, **4**–**9** are non-cytotoxic against DLD-1 cells at the dose up to 20 μM. Compound **2** was weakly cytotoxic: the decrease in cell viability was 20% at 20 μM. The concentration of compound **3** that caused a 50% reduction in cell viability (IC_50_) of DLD-1 cells was 17 ± 0.2 μM. DLD-1 cells possessed 100% viability after treatment with 5 μM concentration of compounds.

### 3.3. Inhibitory Potency of Aaptamine and Makaluvamine Classes of Alkaloids on the α-NaGalase of Cancer Cells

**Figure 1:**
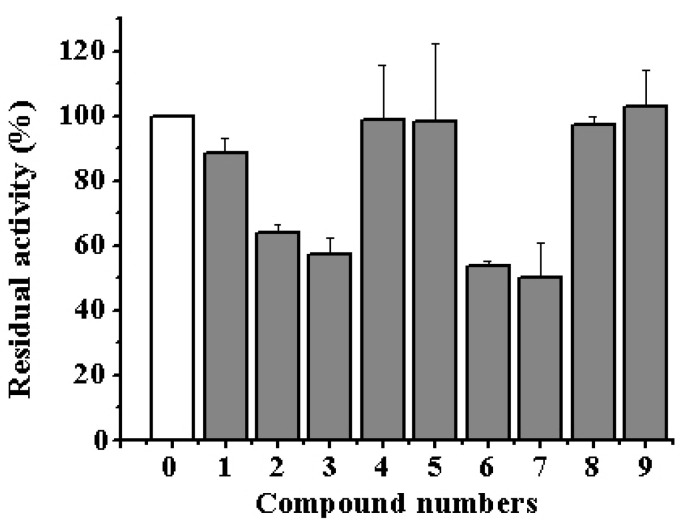
Residual activity of lysate cell α-NaGalase after treatment of colon cancer cells DLD-1 with 5 μM of aaptamine (**1**), isoaaptamine (**2**), 9-demethylaaptamine (**3**), aaptanone (**4**), damirone A (**5**), damirone B (**6**), makaluvamine H (**7**), makaluvamine G (**8**), zyzzyanone A (**9**) relative to the enzyme of untreated cells as positive control (**0**). Residual activity of α-NaGalase was defined as A/A_0_ × 100 (%), where A and A_0_ are the specific activities of α-NaGalase of the samples (**1**–**9**) and the control experiment (**0**), respectively. Data are shown as means ± standard deviation (SD) of values from three independent experiments (*p* < 0.05) evaluated by methods of descriptive statistics in software “Origin 8.1”.

All alkaloids **1**–**9** were tested on their influence on the hydrolyzing activity of the α-NaGalase isolated from cells of human carcinoma DLD-1, HT-29 and HCT-116, breast cancer MDA-MB-231, melanoma SK-MEL-5 and RPMI-7951, and mouse healthy epidermal cells JB6 Cl41. Our experiments showed that none of the tested compounds **1**–**9** had any direct inhibitory effects on the activity not only against human cancer cell α-NaGalase as well against α-NaGalase from mouse healthy epidermal cells. However, isoaaptamine (**2**), 9-demethylaaptamine (**3**), damirone B (**6**) and makaluvamine H (**7**) reduced the expression of the enzyme in the human colorectal adenocarcinoma cell line DLD-1 from 100% to 64, 57, 52, and 50%, respectively, at concentration 5 μM (Figure 1).

We observed a similar effect after treatmant with DLD-1 cells with sulfated polysaccharide fucoidan from brown alga *Fucus evanescens* [37]. Fucoidan significantly reduced the expression of the enzyme, but did not directly inhibit it. Currently, the information about correlation of activity or expression of α-NaGalase with activation of receptors or proteins of signaling pathways, responsible for carcinogenesis, is absent in the literature.

### 3.4. Inhibitory Potency of Aaptamine and Makaluvamine Classes of Alkaloids on the α-PsGal of the Marine Bacterium

According to the modern classification of carbohydrate active enzymes (CAZy), the α-PsGal belongs to the GH36 family and α-NaGalase from human cancer cells belongs to the GH27 family. These enzymes of the GH27 and GH36 families are related, they share a common gene and form clan D, the structure of their active center is very homologous [28]. Accordingly, we decided to test whether alkaloids will inhibit an marine bacterial enzyme closely related. We hoped that effective α-PsGal inhibitors would inhibit the highly malignant α-NaGalase enzyme in cells. All alkaloids **1–9** were tested on the hydrolyzing activity of the α-PsGal isolated from a marine bacterium *Pseudoalteromonas* sp. KMM 701. There was no notable activity against α-PsGal for aaptamine (**1**), aaptanone (**4**), damirones A (**5**) and B (**6**), and makaluvamine H (**7**) even at a high concentration of inhibitors (Table 1).

The tested alkaloids to varying degrees inhibited the GH36 α-PsGal. It is interesting to note that pentacyclic guanidine alkaloids from the sponge *Monanchora pulchra* inhibited only marine microbial enzymes (the α-PsGal and 1,3-exo-β-D-glucanase from marine fungus *Chaetomium indicum*), but did not act on eukaryotic 1,3-endo-β-D-glucanase from marine mollusk *Spisula sacchalinensis* and the GH109 family α-NaGalase from marine bacteria *Arenibacter latericius* KMM 426^T^ [40,52].

Of all tested alkaloids, only four compounds showed any inhibitory effects on the activity of the bacterial α-PsGal. They are isoaaptamine (**2**), 9-demethylaaptamine (**3**), makaluvamine G (**8**), and zyzzyanone A (**9**) (Table 1). It was shown that inhibitors **2**, **3**, **8,** and **9** irreversibly inactivate the α-PsGal. The irreversible inhibition was confirmed by the dialysis experiment. The activity of the enzyme did not recover after dialysis against the buffer solution for 60 h, but the enzyme in the absence of the inhibitor keeps 100% activity during this dialysis process.

### 3.5. Kinetic Studies on **2**, **3**, **8**, and **9** against the α-PsGal

**Figure 2:**
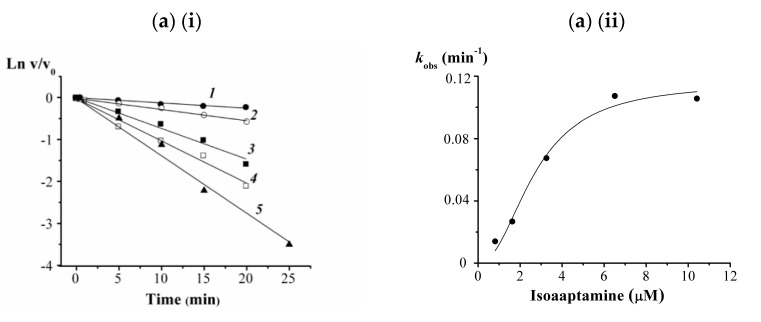

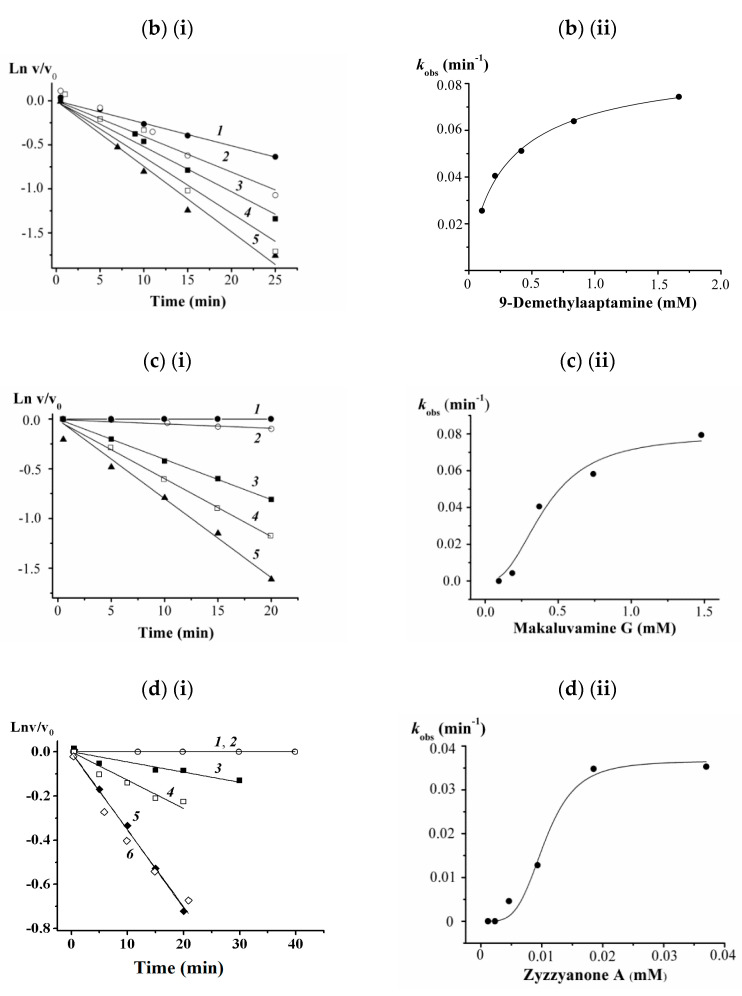
The results of kinetic studies of the α-PsGal inactivation by tested alkaloids: (**i**) the kinetic change of the residual activity of the enzyme (*v/v*_0_) in semilogarithmic coordinates at the presence of different concentrations of alkaloids; (**ii**) the inactivation rate (*k*_obs_) dependence on the concentration of alkaloids; (**a**) isoaaptamine: 814 μM (*1*), 1.63 μM (*2*), 3.255 µM (*3*), 6.51 µM (*4*), 10.417 µM (*5*); (**b**) 9-demethylaptamine: 0.104 mM (*1*), 0.208 mM (*2*), 0.417 mM (*3*), 0.833 mM (*4*), 1.67 mM (*5*); (**c**) makaluvamin G: 0.093 mM (*1*), 0.185 mM (*2*), 0.37 mM (*3*), 0.74 mM (*4*), 1.48 mM (*5*); (**d**) zyzzyanone A: 0.0116 mM (*1*), 0.023 mM (*2*), 0.046 mM (*3*), 0.093 mM (*4*), 0.185 mM (*5*), 0.37 mM (*6*). All of the experiments were performed in duplicates.

To investigate a possible mode of interaction of these alkaloids with the α-PsGal, kinetic studies were done. The results of kinetic studies of the α-PsGal inactivation by the action of the spongean alkaloids **2**, **3**, **8,** and **9** are shown in Figure 2. Because of the α-PsGal inactivation developed relatively slowly, within a few minutes under experimental conditions, the inhibitory activity of the compounds can be described by the inactivation rate constant (*k*_inact_, min^–1^) and equilibrium inhibition constant *K_i_* [53]. The kinetic Equation (1) describes the irreversible slow inhibition of the α-PsGal under the action of alkaloids **2**, **3**, **8**, and **9**:

(1) $$\begin{array}{c} \;K_{i}\;k_{\mathrm{inact}}\\ E+\mathrm{In}\;⇌\left[E\cdot I^{n}\right]\to \;E-I^{n} \end{array}$$

where E is enzyme; I is inhibitor; n is Hill’s coefficient of cooperativity.

Alkaloids **2**, **3**, **8,** and **9** exhibited a time- and concentration-dependent inhibitory manner suggesting that the inhibition was irreversible. The curves of the dependences of the residual activity *v*/*v*_0_ on the time in semilogarithmic coordinates are shown in Figure 2(i) (a, b, c, d). The inactivation rate constant (*k*_obs_), as can be seen in Figure 2(ii) (a, b, c, d) is inhibitor concentration dependent and a gradual increase in inhibitor concentration increases the inactivation rate constant. The nonlinear S-shaped curves of *k*_obs_ dependence on a concentration of compounds **2**, **3**, **8,** and **9** (Figure 2(ii)) mean that inhibition of the α-PsGal by these slowly binding irreversible inhibitors is cooperative and occurs in two stages: (1) the formation reversible enzyme-inhibitor complex (E·I) and (2) irreversible inhibition of the enzyme in this complex (E–I). The experimental dependences of *k*_obs_ on the concentration of the compounds **2**, **3**, **8,** and **9** (Figure 2(ii)) more exactly are approximated by the Hill’s Equation (2). Values of true *K_i_* and *k*_inact_, as well as Hill’s cooperativity coefficients are given in Table 2.

(2) $$k_{\mathrm{obs}}=\frac{k_{\mathrm{inact}}{\;I}^{n}}{\left(\;K_{i}^{n}+{\;I}^{n}\right)}$$

Of the group aaptamine alkaloids, only isoaaptamine (**2**) and 9-demethylaaptamine (**3**) showed inhibitory activity against the α-PsGal. As can be seen in Table 2 isoaaptamine (**2**) (*K_i_* = 2.7 µM) is significantly more active if compared with 9-demethylaaptamine (**3**) (*K_i_* = 300 µM), although their inactivation rate constants are almost equal (*k*_inact_ 0.12 and 0.092 min^−1^, respectively). More active inhibitor **2** shows positive cooperative binding with α-PsGal with n > 1 (*n* = 2.17), i.e., binding of one molecule of **2** with the enzyme increases enzyme affinity for other molecules of this inhibitor. In contrast, less active **3** displays negative cooperative binding with n < 1 (*n* = 0.84); binding of one molecule of **3** decreases enzyme affinity for other molecules of **3**. Structurally, these inhibitors differ only slightly, compound **3** is the non-N1-methylated analogue of compound **2**. A substitution pattern in a benzenoid part of compounds **2** and **3** is the same; both inhibitors have a hydroxyl group at C-9 position in contrast to compounds **1** and 4. The increased activity of **2** in comparison with **3** is presumably the consequence of the presence of the methyl group at N1 of a 1,6-naphthiridine core. Thus, the presence of the hydroxyl group at C-9 and the methyl group at the N-1 in a benzo-1,6-naphthiridine core are important factors for α-PsGal inhibition. Such observation was reported earlier for **2**, which showed higher activity in inhibition of sortase A, compared with **1** and **3** [18]. Isoaaptamine (**2**) also was more active than aaptamine (**1**) in inhibiting β-1,3-glucanase of marine mollusks [20]. Small structural differences in aaptamine (**1**) and aaptanone (**4**) (Table 2) in comparison with **2** and **3** resulted in loss of their inhibitory potency on the α-PsGal.

Among the test alkaloids of the makaluvamine group, makaluvamine G (**8**) and zyzzyanone A (**9**) showed low inhibitory activity against the α-PsGal. Nonetheless, zyzzyanone A (**9**) (*K_i_* = 105 µM) is more active than makaluvamine G (**8**) (*K_i_* = 411 µM), but inactivation rate of **9** (0.037 min^−1^) is almost twice lower that of **8** (0.079 min^−1^) (Table 2). The methylaminoethyl side chain and/or rigid planar dipyrroloquinone core of **9**, apparently, slowly penetrate into the active site of α-PsGal reducing the rate of enzyme inactivation. More active inhibitor **9** shows higher coefficient of cooperativity (n = 4.1) than less active inhibitor **8** (n = 2.5). Comparing structures of alkaloids from the makaluvamine group it can be seen that both metabolites **8** and **9** have a hydroxyphenyl ring in their structure. Probably, the increased electron density of the hydroxyphenyl ring would facilitate non-covalent π–π interactions of these compounds with protein residues in the catalytic or allosteric sites of the enzyme. Earlier it has been shown that pentacyclic guanidine alkaloids from the sponge *Monanchora pulchra* also are slow-binding irreversible inhibitors of the α-PsGal without the formation of covalent bonds with amino acids of the enzyme active center [40].

### 3.6. Theoretical Models of α-D-Galactosidase Complexes with Aaptamine and Makaluvamine Alkaloids

To support the active-site-directed nature of the α-D-galactosidase inactivation and assess possible binding sites for inhibitors a molecular docking of the active alkaloids **2**, **3**, **8,** and **9** was performed on the predicted 3D structure of the α-D-galactosidase active center [54]. Homology model of the α-PsGal 3D-structure was constructed earlier by molecular docking in the MOE program [54] using the crystal structure of the α-galactosidase from *Lactobacillus acidophilus* of GH36 family as template [55]. The active center of the α-D-galactosidase is located in the central (β/α)_8_ domain, Asp451 and Asp516 are the catalytic nucleophile and acid/base residues, respectively. Another highly conserved residue in the active center is Trp308. The enzyme α-PsGal is a typical O-glycoside hydrolase of the GH36 family. It was previously shown that its molecule consists of two identical subunits [29,30]. The computer model of the enzyme has previously been successfully used for the analysis of α-PsGal complexes with low molecular weight inhibitors [39,40,54].

**Figure 3:**
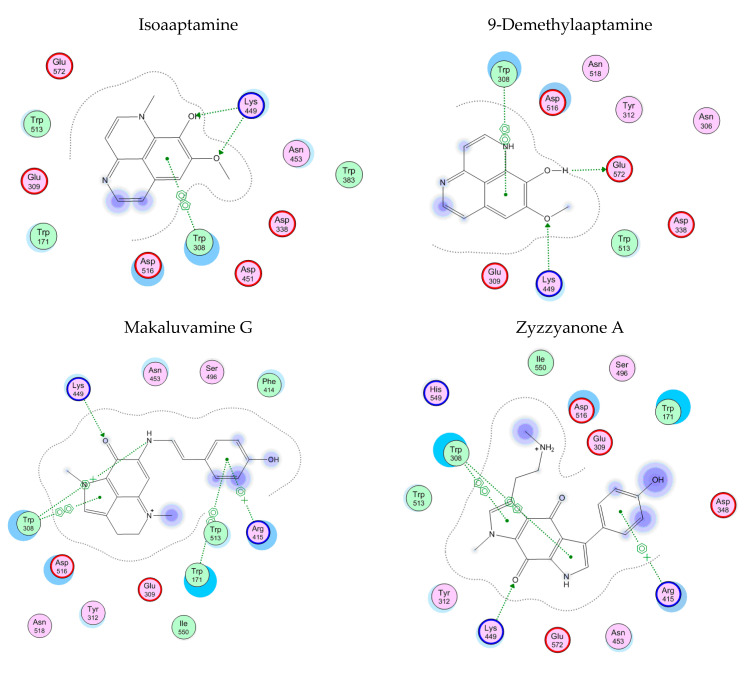

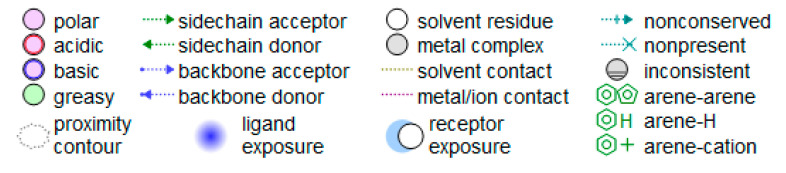
2D-diagrams of the α-PsGal complexes with alkaloids of aaptamine and makaluvamine classes.

**Figure 4:**
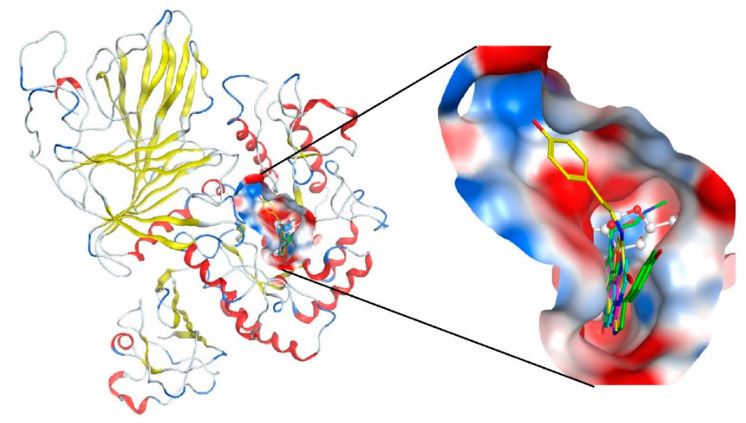
Superpositions of alkaloids **2**, **3**, **8, 9,** and D-galactose in the active site of α-D-galactosidase. Ligands are shown as sticks. Isoaaptamine (**2**) is shown in pink; 9-demethylaaptamine (**3**) is shown in cyan; makaluvamine G (**8**) is shown in yellow; zyzzyanone A (9) is shown in green; D-galactose is shown in white. The molecular surface of the catalytic domain binding site is shown in red (negative), blue (positive), and gray (neutral) classes.

Molecular docking of the alkaloids isoaaptamine (**2**), 9-demethylaaptamine (**3**), makaluvamine G (**8**), and zyzzyanone A (**9**) with α-PsGal showed that all studied compounds form complexes with the active site of the enzyme. The key interactions of these inhibitors with the active site of the α-D-galactosidase are shown in Figure 3. The complexes of the alkaloids with the α-PsGal active center are stabilized by π–π interactions with functionally important residue Trp308. Moreover, all alkaloids form H-bonds with residue Lys449. As can be seen from superposition of complexes of compound **2**, **3**, **8**, **9,** and D-Gal with the active center of the enzyme (Figure 4), the alkaloids shared space in the active center with D-Gal. This indicates the active-site-directed nature of the α-D-galactosidase inactivation by the tested compounds. Although the alkaloids **2**, **3**, **8**, and **9** did not directly interact with catalytic residues Asp451 and Asp516, tricyclic parts of alkaloids located deeply in the active center block the access of the substrate into the pocket of the active center to the catalytic residues Asp451 and Asp516.

**Figure 5:**
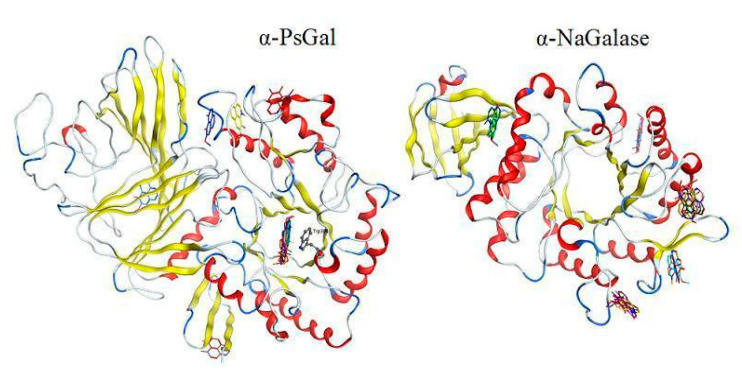
Superposition of the 20 most beneficial complexes of isoaaptamine with one subunit of bacterial α-D-galactosidase of GH36 family and human lysosomal α-NaGalase (Protein Data Bank: 3H54) of GH27 family. Protein structures are shown in ribbon diagrams, the isoaaptamine molecule is shown as sticks. The structures of the complexes were calculated using the GRAMM program.

None of the studied alkaloids formed complexes with the active site of human α-NaGalase. If, in the case of α-PsGal, isoaaptamine binds to the active site of the enzyme, then, in the case of α-NaGalase, binding sites of the alkaloid are located outside the active site of the enzyme (Figure 5). Therefore, bound isoaaptamine has not decreased the activity of cancer α-NaGalase.

## 4. Conclusions

We used two related α-glycosidases of Clan D: α-N-acetylgalactosaminidase (α-NaGalase) of the GH27 family from different human cancer cells and α-galactosidase (α-PsGal) of the GH36 family from the marine bacterium *Pseudoalteromonas* sp. KMM 701 to study the effect of the well-known marine sponge metabolites with a therapeutic potential and clarify the possible mechanism of their inhibitory action. The well-characterized α-PsGal has been proved to be a suitable model for characterizing the novel properties of the alkaloids. Isoaaptamine (**2**), 9-demethylaaptamine (**3**), makaluvamine G (**8**), and zyzzyanone A (**9**) have been shown to be irreversible slow-binding inhibitors of the α-PsGal of the GH36 family. The inhibitory ability of these alkaloids depends on the chemical structure of their molecules. The presence of the hydroxyl group at C-9 position and the N1-methyl group on a benzo-1,6-naphthyridine skeleton are important factors for α-PsGal inhibition by aaptamine alkaloids. A hydroxyphenyl ring in makaluvamine G and zyzzyanone A is important structural feature for their activity against the α-PsGal. Our findings have demonstrated the potential of marine natural products to be a prototype of the α-galactosidase inhibitors. However, these highly active marine compounds act selectively on carbohydrases; they have no direct inhibitory effect on the activity of the cancer α-NaGalase of the GH27 family. Therefore, the expected properties, similar to those of α-PsGal, in the related eukaryotic enzyme (α-NaGalase) were not confirmed. It is interesting to note that the same compounds isoaaptamine (**2**), 9-demethylaaptamine (**3**), and two other compounds damirone B (**6**) and makaluvamine H (**7**) reduced the expression of the enzyme in the cell line DLD-1 at non cytotoxic concentration 5 μM. However, further in vitro and in vivo studies are needed to study the mechanism of molecular suppression of α-NaGalase in cancer cells.

## Figures and Tables

**Table 1:** Residual activity *v/v*_0_ (%) of the α-PsGal after incubation with alkaloids **1–9.**

Compound	*v/v*_0_ (%)	Compound	*v*/*v*_0_ (%)
**1**	79 ^1^	**6**	87 ^2^
**2**	0 ^1^	**7**	67 ^2^
**3**	0 ^1^	**8**	28 ^2^
**4**	100 ^1^	**9**	0 ^2^
**5**	87 ^2^	6.4% EtOH	100

^1^ Compound concentrations 1.67 mM; ^2^ compound concentrations 1.48 mM; the enzyme was preincubated with an inhibitor for 30 min, 20 °C, pH 7.0.

**Table 2:** Structures of tested spongean alkaloids and α-PsGal inhibition constants for **2**, **3**, **8,** and **9.**

Compound	Structure	*K_i_* (µM) ^1^	*k*_inact_ (min^−1^) ^2^	n ^3^
aaptamine (**1**)	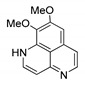	nd ^4^		
isoaaptamine (**2**)	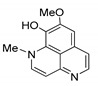	2.70 ± 0.42	0.12 ± 0.01	2.17 ± 0.55
9-demethylaaptamine(**3**)	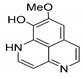	300 ± 77	0.092 ± 0.008	0.84 ± 0.12
aaptanone (**4**)	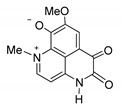	nd ^4^		
damirone A (**5**)	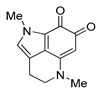	nd ^4^		
;damirone B (**6**)	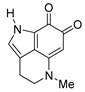	nd ^4^		
makaluvamine H (**7**)	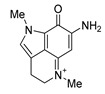	nd ^4^		
makaluvamine G (**8**)	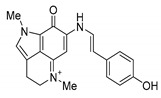	411 ± 86	0.079 ± 0.011	2.5 ± 0.97
zyzzyanone A (**9**)	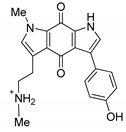	105 ± 9	0.037 ± 0.003	4.10 ± 1.42

^1^ *K_i_*-equilibrium inhibition constant; ^2^ *k*_inact_-inactivation rate constant; ^3^ n-Hill’s coefficient of cooperativity; ^4^ not determined.

## Data Availability

Not applicable.
